# Clinical routines and structural resources for performing transoesophageal echocardiography on German stroke units

**DOI:** 10.1186/s42466-026-00500-9

**Published:** 2026-05-19

**Authors:** Damjan Mirkov, Adeeb Qabalan, Peter A. Ringleb, Stefan Schwab, Karl Georg Haeusler, Ekkehart Jenetzky, Timolaos Rizos

**Affiliations:** 1 Department of Neurology, University of Heidelberg, Im Neuenheimer Feld 400, 69120 Heidelberg, Germany; 2 Department of Neurology, University Hospital Erlangen, Erlangen, Germany; 3 Department of Neurology, University Hospital Ulm, Ulm, Germany; 4 Faculty of Health/School of Medicine, Witten/Herdecke University, Witten, Germany; 5 Department for Child and Adolescent Psychiatry, Johannes Gutenberg- University, Mainz, Germany

**Keywords:** Ischemic stroke, TIA, Transoesophageal echocardiography, Stroke unit, Germany

## Abstract

**Background:**

Transoesophageal echocardiography (TOE) is essential for identifying cardiac sources of embolism in patients with acute ischemic stroke (AIS) or transient ischemic attack (TIA). As randomized controlled trials are missing, recommendations regarding echocardiography after stroke remain vague, and clinical routines and structural resources for performing TOE on stroke units may differ. We here examined structural conditions for performing TOE on certified stroke units in Germany, and evaluated factors that may influence the decision to perform TOEs.

**Methods:**

In this prospective exploratory cross-sectional survey, a standardized anonymous questionnaire was sent to all clinical leads of certified stroke units in Germany, supported by the German Stroke Society. The questionnaire focused on (a) on general characteristics of stroke units and logistics of TOE examinations and (b) local indication for performing TOEs, with a focus on factors that may influence this decision.

**Results:**

Data from 248 SUs (stroke units) were included into the analysis (response rate 71.1%). The reported median TOE rate was 23.0% (IQR 18–34). Lower TOE rates were related to increasing numbers of stroke patients (*p* = 0.048). Most TOEs were performed within 24–48 h after the request (49.6%). Longer waiting times for TOE were associated with level of certification (*p* < 0.001) and higher numbers of stroke patients (*p* = 0.002). The presence of a stroke unit cardiologist did not result into shorter waiting times (*p* = 0.238). In total, 23.8% of the responding stroke units do not have a standardized operating procedure (SOP) for indicating TOE. Though 50% of leads considered quantitative requirements for indicating TOEs, within certain clinical contexts and situations, our findings revealed a broad consensus among stroke unit leads concerning TOE indications.

**Conclusions:**

Despite limitations, our study provides valuable insights into performing TOE in hospitalized German stroke patients. This may facilitate the optimized use of TOE in hospitals and represent a first step towards the development of future guidelines and SOPs, in particular in the context of limited resources.

**Supplementary Information:**

The online version contains supplementary material available at 10.1186/s42466-026-00500-9.

## Introduction

The treatment of patients suffering acute ischemic stroke or transient ischemic attack (TIA) on dedicated stroke units is associated with better outcome after stroke [1]. Acute stroke care in Germany is provided predominantly by stroke units certified by the German Stroke Society (DSG), following distinct certification criteria [2, 3]. To ensure the quality of treatment and care for stroke patients, a comprehensive quality assurance system for the treatment of ischemic stroke and TIA patients has been implemented in Germany [4]. This system includes the standardization of diagnostics, therapy and in-hospital rehabilitation, documentation of treatment procedures and results to improve the quality of care [4].

A major aim of stroke unit treatment is to ascertain the underlying cause of ischemic stroke or TIA to initiate the most appropriate secondary preventive measures. It has been demonstrated that approximately 25% of all ischemic strokes are attributable to cardioembolism [5, 6]. In addition to the most prevalent atrial fibrillation (AF), this encompasses cardiac thrombi due to ventricular dysfunction, valvular disease, persistent patent foramen ovale (PFO), or intracardiac tumors [6, 7]. A comprehensive cardiac evaluation of patients with ischemic stroke and TIA on stroke units is therefore of high significance for secondary stroke prevention. In this context, transoesophageal echocardiography (TOE) is superior to transthoracic echocardiography (TTE) to detect possible cardiac embolic sources [8, 9]. In addition, large aortic plaques can be detected by this method, which is considered to be cost-effective but investigator-dependent [7, 10]. While cardiac computed tomography as well as cardiac magnetic resonance imaging may offer comprehensive non-invasive cardiac evaluation, their use in clinical practice is very low, considering the expense of increased costs or radiation exposure. However, there is no randomized controlled trial (RCT) demonstrating that the choice of the cardiac imaging method influences the prognosis of stroke patients. Therefore, guideline recommendations remain vague [10] and the extent of TOE examinations on German stroke units varies considerably [11].

To the best of our knowledge, clinical routines and structural resources for performing TOE on German stroke units have not yet been described. Furthermore, there are no data on the structural conditions that may influence the use of TOEs, which may help to inform future guidelines. To close this gap, we examined the structural conditions for performing TOE on certified stroke units in Germany, and evaluated factors that influence the decision to perform TOE.

## Materials and methods

### Study design

This prospective exploratory cross-sectional study was conducted in May 2024 by an anonymous survey addressing the clinical leads of all stroke German stroke units certified by the German Stroke Society (Deutsche Schlaganfall-Gesellschaft; DSG) [3] (*n* = 349). Stroke unit (SU) certification in Germany is divided into “regional” SUs (representing “primary stroke centers”), “supraregional” SUs (representing comprehensive stroke centers, offering the entire range of neurological, neuroradiological, and neurosurgical services) [12] and telemedically-connected SUs (primary stroke services without 24/7/365 presence of neurologists) [3, 13].

A standardized questionnaire was sent by the DSG to the medical leads of all certified stroke units by post. To improve the response rate, all leads received a reminder to return the questionnaire by post two weeks later. All returned copies of the questionnaire were checked for probable misinterpretations, comments and unclear answers before they were accepted for analysis. Anonymized paper-based case report files (CRF) were entered into an electronic database.

### Questionnaire

The questionnaire was divided into two parts (cf. Supplementary Material). The first included questions on the general characteristics of the SU, including the number of beds, treated stroke patients per year, and the logistics of TOE examinations (i.e. presence of a cardiology department, presence of standard operating procedures (SOPs), and waiting times until TOE). The second part referred to indications for performing a TOE, focusing on demographic, clinical and imaging conditions. Stroke unit certification follows definded criteria of the German Stroke Society (DSG). The certification process includes reporting of TOE rates in ischemic stroke/TIA patients during hospitalization. For this study, data reported in 2023 were analysed. To enable a detailed analysis regarding the influence of each factor on the indication to perform a TOE, all responses were pre-structured using a six-point Likert scale ranging across the following categories: never (0%), sporadically (< 25%), occasionally (25–50%), frequently (50–75%), regularly (75–99%) and always (100%).

The survey was performed in a completely anonymous manner. The identity of the participating hospital or the responding clinical lead within the hospital was unknown. As no individual patient level data were collected, informed consent was waived. The study was performed in accordance with the principles of the Declaration of Helsinki. Approval from the local ethics committee to conduct the study (S-087/2024) was obtained. The manuscript was developed according to the STROBE guidelines for reporting secondary data [14].

### Statistical analysis

The descriptive data collected were presented as absolute and relative frequencies, while the ordinal and continuous data were presented as medians and interquartile ranges (IQRs). Differences between groups were examined using univariate nonparametric tests and logistic regression analyses. A two-sided *p* value of < 0.05 was considered as exploratively significant. Data were analysed by using the Statistical Package for the Social Sciences (SPSS 29.0).

## Results

### General characteristics of centers and information on TOE

In total, 248 leads of certified German stroke units participated (response rate 71.1%). In detail, leads of 116 supraregional SUs, 117 regional SUs and 15 telemedically-connected SUs participated. The majority of units were equipped with 9–12 beds (31.5%) and treated 750–999 stroke patients a year (25.8%) (Table 1). A cardiology department on-site was present in 88.3% of centers at the site of the hospital. Stroke unit certification levels were associated with numbers of patients (*p* < 0.001), higher numbers of stroke unit beds (*p* < 0.001) and a higher probability of having a cardiology department on site (*p* = 0.006, Table 2).

**Table 1:** General information and structural characteristics of the participating stroke units (SU)

Characteristics of the participating stroke units	Number (*N*)	Percentage (%)
Level of certification		
Supraregional SU. Regional SU. Telemedically-connected SU.	116 117 15	46.8 47.2 6.0
Number of stroke units’ beds		
≤4. 5 - 6. 7 - 8. 9 - 12. >12.	26 62 54 78 28	10.5 25.0 21.8 31.5 11.3
The total number of patients with ischaemic stroke or transient ischaemic attack (TIA) who were treated in stroke units in the year before the survey (2023)		
<250. 251-500. 501-749. 750-999. 1000-1250. >1250.	9 35 59 64 53 28	3.6 14.1 23.8 25.8 21.4 11.3
The presence of a cardiology department		
Cardiology department present. Without cardiology department.	219 29	88.3 11.7
Transoesophageal echocardiography (TOE) is carried out by:		
Cardiology department. Medical practice or medical care center (MVZ) during the hospital stay. Medical practice or medical care center (MVZ) owned by the hospital. Cardiologist on the stroke unit. Medical practice or medical care center (MVZ) after discharge. External medical practice or medical care center. Internal medicine department.	212 2 2 21 1 2 8	85.5 0.8 0.8 8.5 0.4 0.8 3.2
Waiting time (in hours) from registration to performance of the transoesophageal echocardiography (TOE)		
<12 h. 12–24 h. 24–48 h. >48 h.	9 75 123 41	3.6 30.2 49.6 16.5
Presence of an in-house standards of operating procedure (SOP) for the indication of transoesophageal echocardiography (TOE) examinations in stroke/TIA patients		
SOP exists. Without SOP.	189 59	76.2 23.8
Are transoesophageal echocardiography (TOE) results helpful for clinical decisions?		
Sporadically (<25%) Occassionally (25-50%). Frequently (50-75%). Regularly (75-99%). Always (100%).	29 71 83 56 9	11.7 28.6 33.5 22.6 3.6
The indication for transoesophageal echocardiography (TOE) examinations often has to be negotiated vigorously with TOE providers in individual patients		
Yes. No.	48 200	19.4 80.6
Do quantitative specifications (e.g. SU certification) influence your indication for a TOE?		
Yes. No.	114 134	46.0 54.0

**Table 2:** Correlation matrix showing the association between different collected variables

	Level of certification	Total number of ischemic strokes/TIA patients treated per year (2023)	Number of stroke unit beds	Presence of cardiology department	Types of units performing the TOE	Waiting time to TOE performance	Existence of an in-house SOP	TOE results helpful for clinical decisions	Indication for TOE need to be negotiated vigorously with TOE providers	Quantitative specifications influence the indication for a TOE	TOE rate
Level of certification		<0.001^#L^	<0.001^F^	0.006^#^	0.115^F^	<0.001^F^	0.176^#^	0.848^#^	0.313^#^	0.978^#^	0.439*
**Total number of ischemic strokes/TIA patients treated per year (2023)**	**<0.001** ^**#L**^		**<0.001** ^**F**^	0.072^F^	**0.004** ^**F**^	**<0.001** ^**F**^	0.154^#L^	0.353^F^	0.152^#L^	0.498^#L^	0.048*
**Number of stroke unit beds**	**<0.001** ^**F**^	**<0.001** ^**F**^		**<0.001** ^**#L**^	**0.007** ^**F**^	**0.002** ^**F**^	**0.036** ^#**L**^	0.651^F^	**0.019** ^#**L**^	0.541^#L^	0.060*
**Presence of cardiology department**	**0.006** ^**#**^	0.072^F^	**<0.001** ^**#L**^		**<0.001** ^**F**^	0.305^#L^	**0.013** ^**#**^	0.387^F^	0.511^#^	**0.013** ^**#**^	0.383*
**Types of units performing the TOE**	0.115^F^	**0.004** ^**F**^	**0.007** ^**F**^	**<0.001** ^**F**^		0.135^F^	0.136^F^	0.534^F^	0.914^F^	**0.002** ^F^	0.724*
**Waiting time to TOE performance**	**<0.001** ^**F**^	**<0.001** ^**F**^	**0.002** ^**F**^	0.305^#L^	0.135^F^		**0.026** ^#**L**^	0.848^F^	**0.041** ^#**L**^	0.069^F^	0.232*
**Existence of an in-house SOP**	0.176^#^	0.154^#L^	**0.036** ^#**L**^	**0.013** ^**#**^	0.136^F^	**0.026** ^#**L**^		0.287^#L^	0.949^#^	0.904^#^	0.760*
**TOE results helpful for clinical decisions**	0.848^#^	0.353^F^	0.651^F^	0.387^F^	0.534^F^	0.848^F^	0.287^#L^		0.188^#L^	**<0.001** ^**#L**^	0.003*
**Indication for TOE need to be negotiated vigorously with TOE providers**	0.313^#^	0.152^#L^	**0.019** ^#**L**^	0.511^#^	0.914^F^	**0.041** ^#**L**^	0.949^#^	0.188^#L^		**0.004** ^**#**^	0.228*
**Quantitative specifications influence the indication for a TOE**	0.978^#^	0.498^#L^	0.541^#L^	**0.013** ^**#**^	**0.002** ^F^	0.069^F^	0.904^#^	**<0.001** ^**#L**^	**0.004** ^**#**^		<0.001*
**TOE rate**	0.439*	0.048*	0.060*	0.383*	0.724*	0.232*	0.760*	0.003*	0.228*	<0.001*	
**Stroke unit cardiologist**	**0.032** ^**#**^	**0.005** ^**F**^	0.069^**F**^	**<0.001** ^**#**^		0.194^**F**^	0.589^**F**^	0.710^**F**^	0.383^**F**^	0.396^#^	0.196*

Legend: * - Student’s t-test; ^#^ - Chi-squared test; ^#L^ - Chi-squared test (Linear by Linear Association), ^F^ - Fisher’s Exact Test, TOE – transoesophageal echocardiography; SOP – standard of operating procedures; TIA – transitory ischemic attack

### Conducting TOE

Standardized operating procedure (SOP) for echocardiography were present in 189 of the responding stroke units (76.2%, Table 1) and were associated with to the local presence of a cardiology department (*p* = 0.013, Table 2). Most TOEs were conducted by the staff of the cardiology department in the respective hospitals (85.5%), followed by SU cardiologists (8.5%, Table 1). The presence of an SU cardiologist (*N* = 21̸248) was associated with higher certification levels (*p* = 0.032) and higher number of treated patients (*p* = 0.005, Table 2).

**Fig. 1:**
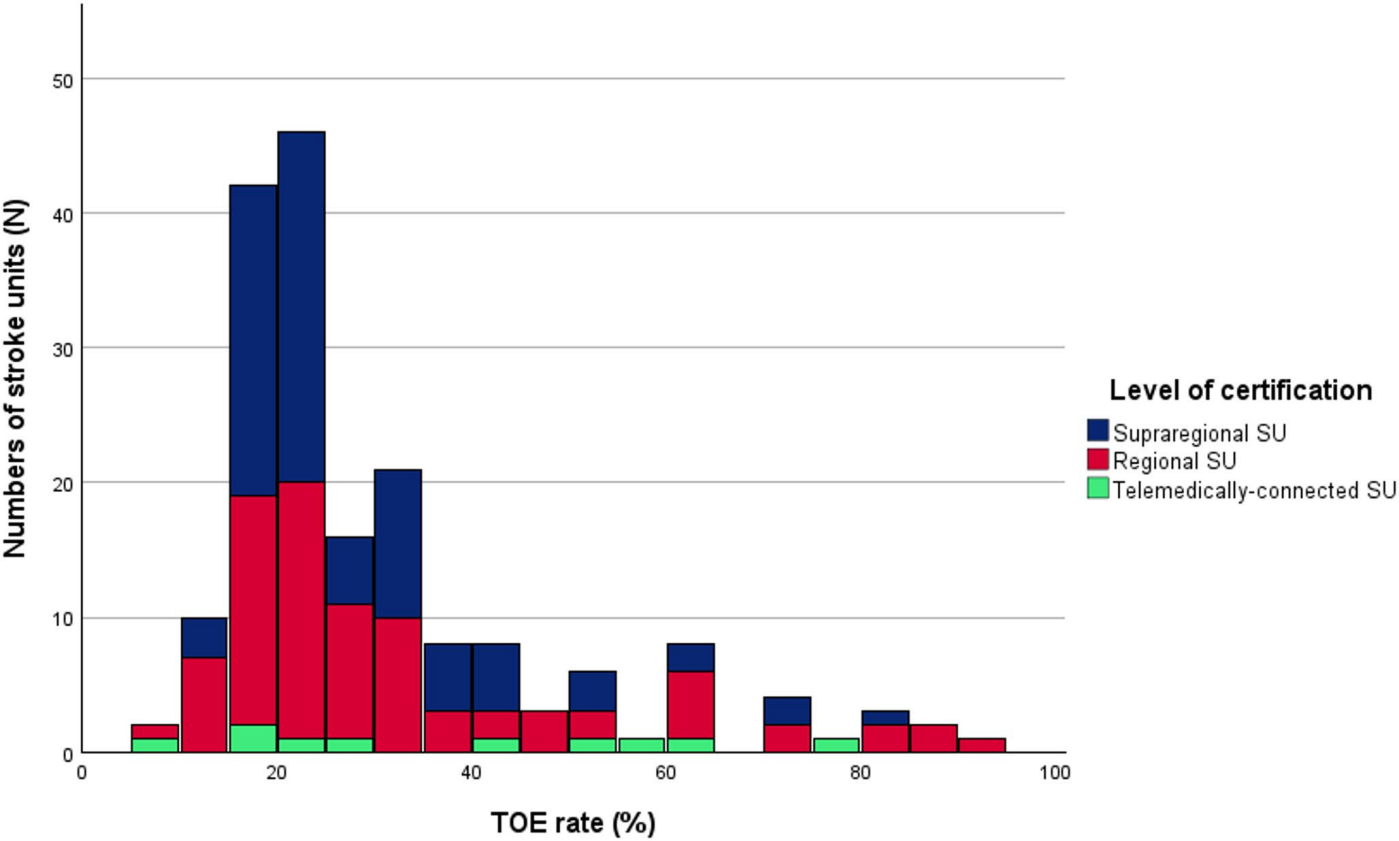
Histogram representing the distribution of TOE rates of all participating centers; TOE – transoesophageal echocardiography

**Fig. 2:**
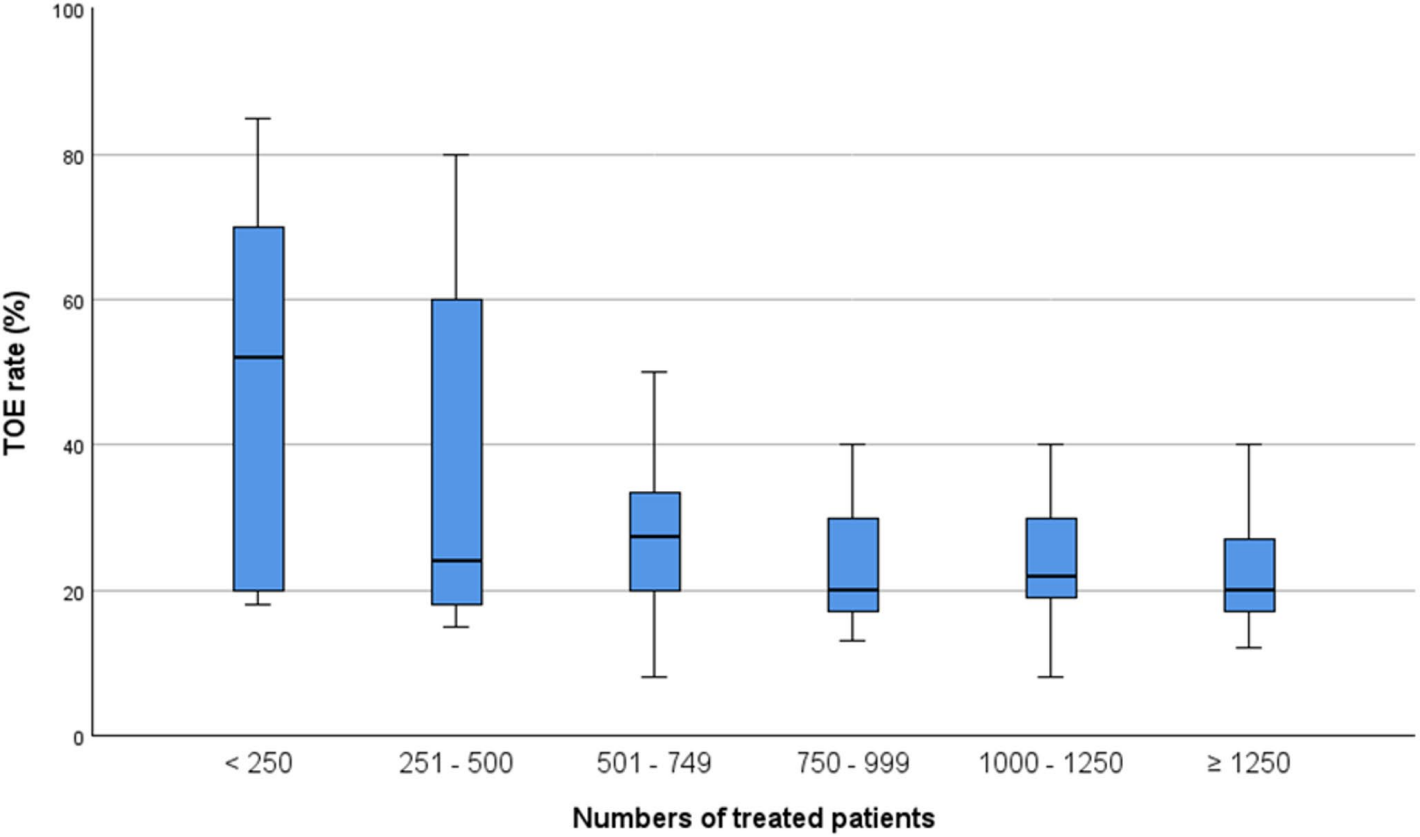
The relationship between the TOE rate and the numbers of treated patients; TOE – transoesophageal echocardiography

The median TOE rate was 23.0% (IQR 18.0 and 34.0, Fig. 1). No differences between certification levels and TOE rates were present (*p* = 0.439, Table 2) but higher numbers of treated stroke patients resulted into lower TOE rates (*p* = 0.048, Fig. 2; Table 2). No association between TOE rates and the presence of an SU cardiologist was observed (*p* = 0.196, Table 2). The results of the binary logistic regression between TOE categories did not show a statistically significant impact of the independent variables on the TOE rate (Supplementary Tables 4a-c).

According to the SU leaders’ estimation, the TOE was performed most frequently 24–48 h after the request (49.6%, Table 1). Prolonged latencies until the TOE was performed were associated with higher levels of SU certification and higher numbers of patients (*p* < 0.001 and *p* < 0.001 respectively, Table 2 and Supplementary Fig. 1). The presence of SU cardiologists did not result into shorter latencies until TOE (*p* = 0.194, Table 2). Binary logistic regression regarding the time until the start of the TOE examination revealed that a waiting time > 48 h was associated with a higher number of treated patients (Supplementary Tables 5a-c).

Focusing on stroke units with ≤ 12 beds revealed that it was increasingly often reported by leads that the indication for TOE examinations has to be negotiated vigorously with TOE providers (*p* = 0.047, Supplementary Fig. 2).

Overall, 46.0% of leads responded that quantitative requirements influence TOE indications (Table 1). This response was more frequent in case when no cardiological department was present (*p* = 0.013) and in cases where an increased frequency of vigorously negotiations by stroke unit leads and TOE providers about TOE indications in individual patients were reported (*p* = 0.004, Table 2).

**Fig. 3:**
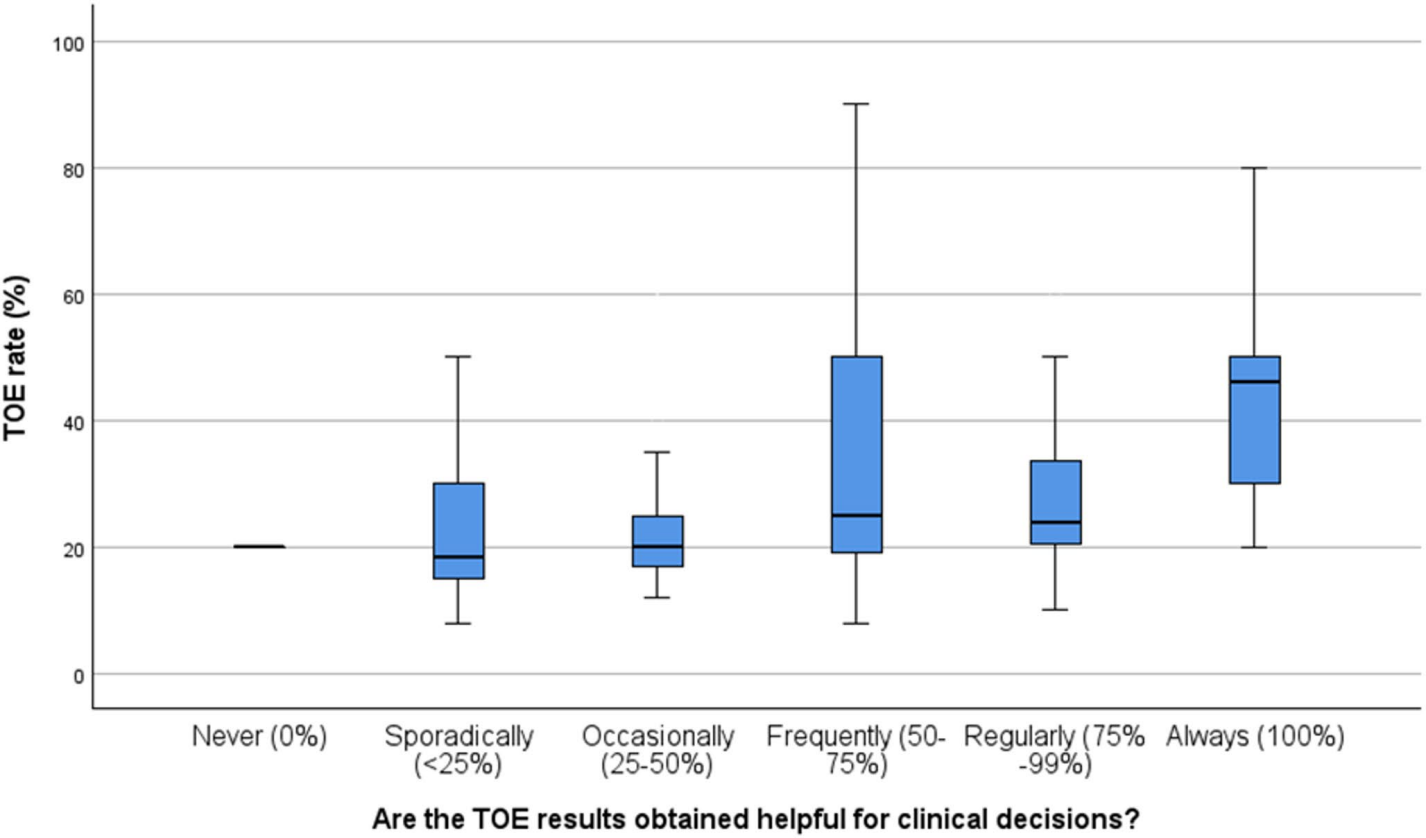
TOE rates and classified helpfulness for clinical decisions by stroke unit leads; TOE – transoesophageal echocardiography

If TOE results were overall considered less helpful for clinical decisions, respondents more frequently indicated that quantitative requirements influence TOE indications (*p* < 0.001). On the other hand, the more helpful TOE results were considered for clinical decisions, TOE rates significantly increased (*p* = 0.002, Fig. 3).

### Specific indications for TOE

**Fig. 4:**
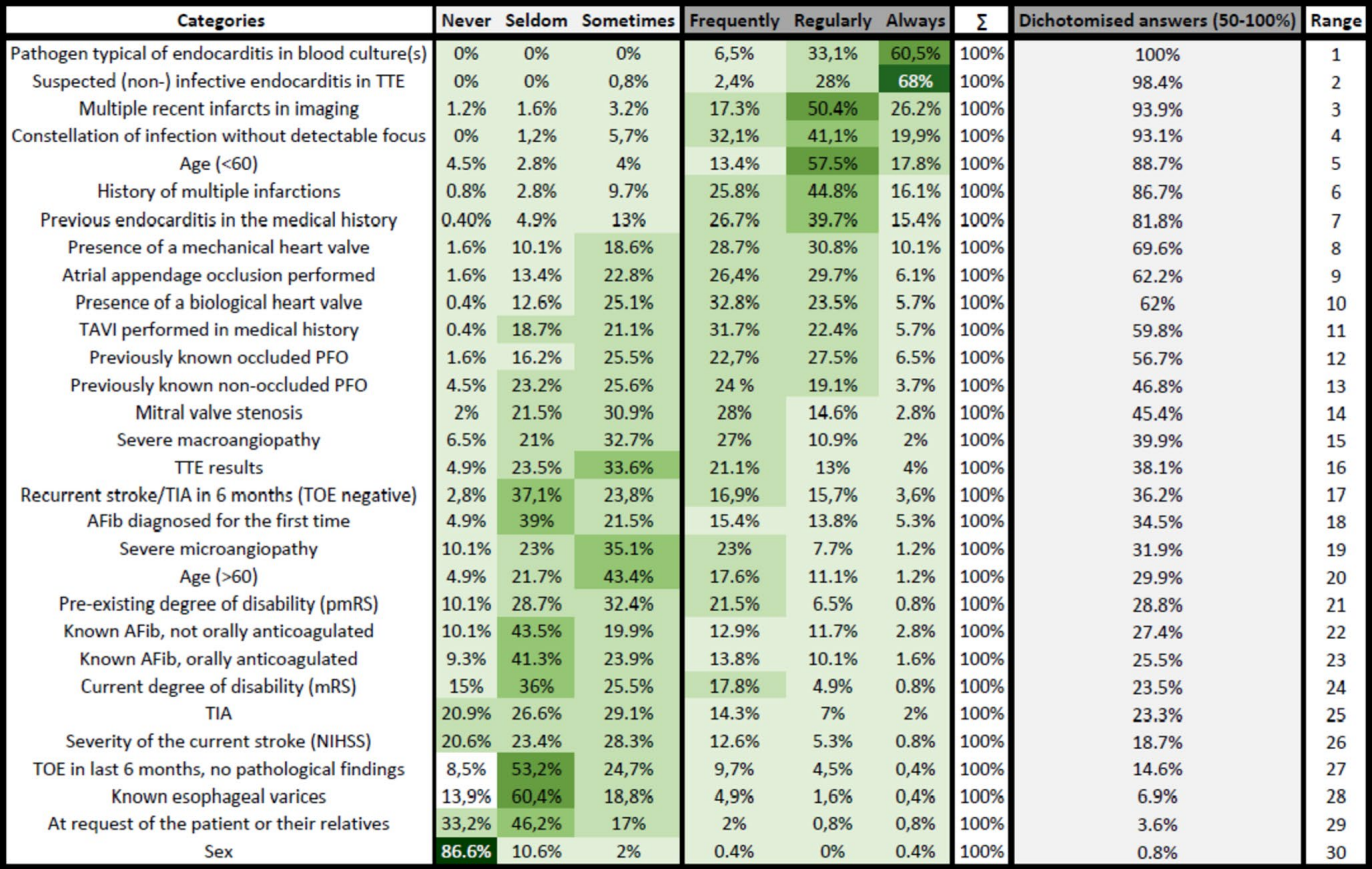
Impact of different findings on the decision to perform transoesophageal echocardiography (TOE) in stroke units; The dichotomized answers include all responses marked as ‘frequently,’ ‘regularly,’ or ‘always’ (representing 50–100% in the questionnaire). These dichotomized values are moreover ranked by their importance as provided by the responding stroke unit leads (i.e., 100% of leads reported that a pathogen typical of endocarditis in blood culture(s) should be an indication for TOE)

Figure 4 summarizes all responses regarding the impact of particular demographic, clinical, laboratory, or imaging findings on the individual decision to perform TOE by the participating leaders of certified stroke units in Germany. Moreover, responses were dichotomized, and ranked in order of importance. Almost all stroke unit leads would recommend a TOE for patients with typical endocarditis findings in blood cultures or TTE findings indicating endocarditis (100% and 98.4%, respectively; Fig. 4). Moreover, TOE is indicated by 93.9% and 93.1% of leads in patients with multiple ischemic strokes on brain imaging or in the presence of an infection without a source, and by 88.7% in patients aged under 60 years. In addition, supplementary radar charts (Supplementary Fig. 3) display the significance of all queried clinical and demographic factors for the indication of TOE, as valued by the participating leads.

## Discussion

This prospective cross-sectional survey provides representative insights into clinical routines and structural resources for performing TOE on German stroke units. Moreover, factors that influence performing TOE examinations have been evaluated. This study is country-specific, and to the best of our knowledge, no other study has specifically examined structural conditions for performing TOE in-hospital and evaluated factors that influence the decision to perform TOE on certified stroke units, neither in Germany nor in other countries.

The major findings of our analysis were that (1) there was a marked decrease in TOE rates with increasing numbers of patients treated per year, (2) almost 50% of stroke unit leads considered quantitative requirements with regard to certification procedures to be an influencing factor in the indication for TOE, (3) approximately 40% of leads considered TOE results to be only sporadically or occasionally helpful for clinical decisions, and (4) almost 25% of stroke units did not have a standardized operating procedure (SOP) for indicating TOE examinations. In addition (5), there appears to be a broad consensus among stroke unit leads concerning TOE indications within certain clinical contexts and situations.

Predefined criteria for the certification of German stroke units include a TOE in at least 15% of all stroke patients, which is based on expert consensus [15]. Though some centers obviously perform TOE examinations in > 80% of all stroke/TIA patients, the overall median TOE rate on German stroke units found in the present study was 23% and a marked decrease in TOE rates was observed with increasing numbers of patients treated per site. This was also described in another nationwide study on echocardiography on stroke units in Germany [11]. Similar to TOE rates, the cited study also showed that TTE rates decreased with the number of stroke patients per department. Moreover, an inverse correlation was observed between TTE and TOE: Higher TOE rates were significantly associated with lower TTE rates per site. Although an SU cardiologist is more often present in high volume stroke centers compared to smaller stroke units (*p* = 0.032), we observed no association between TOE rates or waiting times until TOE and the presence of an SU cardiologist. The underlying reasons for these findings remain speculative. Probably, limited resources are responsible for this observation but it would also be conceivable that high volume centers employ TOEs in more specific and constrained clinical scenarios compared to centers with lower numbers of patients. We cannot provide data with regard to the variability of workflow with regard to TOE registration/request in different ala. It is also to note that stroke unit certification criteria by the DSG have been amended after the current study was conducted. At present, TOE rates of 10–15% are accepted, if a local SOP with regard to TOE performance is existent [16]. This adjustment is attributable to the experience that the former 15% criterion was barely met in many cases, despite meticulous case selection with proven well-founded patient selection procedures for TOE examinations in the respective departments. This supports the assumption that rates of TOE are also influenced by patient selection. A feasible solution could be the development of consensus statements of patient-related and disease-specific factors, based on scientific evidence. Such could lead indicating future TOE examinations. However, respective clinical studies are, to our knowledge, not available yet.

The median TOE rate of 23% found in the present study is in line with another study of 310 German stroke units using DSG certification data (TOE rate 21.3%) [11] and mirrors most probably predefined DSG stroke unit certification criteria. This assumption is also reinforced by the finding that nearly half of all stroke unit leads stated quantitative requirements to be an influencing factor when indicating TOE examinations. It is to acknowledge that invasive procedures, which have the potential for harmful side effects, should not be performed on the basis of quantitative requirements for future certifications but on the basis of defined clinical situations.

Interestingly, in patients participating in randomized stroke trials in Germany, reported TOE rates were considerably higher compared to our findings. A sub-analysis of the randomised controlled MonDAFIS trial at certified German stroke units reported a median TOE rate of 54% (IQR: 34–65%) [17], which was 39% (range: 9–80% by centers) in the FIND-AFrandomized trial [18]. As TOE was not a specific study procedure or mandatory in these two stroke trials, one might speculate that these higher rates may indicate that the utilisation of TOE on German stroke units participating in prospective studies may differ from that of other units [17, 18].

No controlled trial has proven that TOE examinations would reduce clinically relevant endpoints in ischemic stroke or TIA patients [10]. In fact, national and international guideline recommendations on the extent of TOE in patients with ischemic stroke and TIA are based on expert consensus and therefore remain vague and contradictory in part [19–24]. Notably, 40% of responding leads stated that TOE results are only sporadically or occasionally helpful for clinical decisions. This assessment is supported by the findings of a recent multicentre prospective observational study that compared TOE and transthoracic echocardiographic (TTE) findings relevant to the further treatment of patients with ischemic stroke [25]. Here, an overall increase of treatment-relevant findings by TOE was reported in 6.4% (29/454 patients). When excluding patients with PFO (*N* = 23/454), the incremental diagnostic yield compared to TTE was only 1.3% (6/454 patients). In patients aged ≤ 60 years (*N* = 191), an increase of treatment-relevant findings by TOE was reported in one out of seven patients. Again, excluding patients with PFO (*N* = 23/191) led to an incremental diagnostic yield by TOE of only 2.1% (4/191 patients) [25].

According to our survey, when stroke units with > 12 beds were excluded, it was observed that the need to negotiate TOE indications with providers was reported more frequently as the number of beds increased. This may not reflect clinically relevant indications but rather the requirement to meet predefined criteria for stroke unit certification.

Taken together, our findings suggest that considerably lower median TOE rates would be recorded if such criteria were not in place. Interestingly, the existing certification criteria do not explicitly delineate which ischemic stroke/TIA patients should or should not undergo TOE [15]. Additionally, both national and international guidelines do not provide clear and comprehensive criteria for identifying patients who would benefit from TOE [21, 26]. Notably, according to our data 25% of stroke units do not have a standardized operating procedure (SOP) guiding the indication for TOE examinations on the level of stroke units. However, the presence of SOPs was not associated with higher TOE rates, or waiting time until TOE examinations. Furthermore, no association between SOPs to the evaluation of leads concerning helpfulness of obtained TOE results for clinical decisions (*p* = 0.287) or to TOE rates (*p* = 0.760) were observed. On the other hand, within certain clinical contexts and situations, our findings revealed a broad consensus among stroke unit leads concerning TOE indications (cf. Supplementary Fig. 3). Among others, these include findings typical for endocarditis, multiple infarcts on recent imaging, a constellation of infection without a detectable focus, age < 60 years and the presence of mechanical heart valves (cf. Fig. 4).

Importantly, overall quotas do not take into account the characteristics of patients on the respective stroke units. For instance, stroke units without the possibility of endovascular treatment on-site might have lower rates of younger patients with embolic stroke patterns. Hence, it would be worth considering more specific guidelines on which specific patients should receive a TOE. Such could encompass demographic and clinical factors, including e.g., age, imaging and laboratory findings.

Besides the reported strengths, our analysis has limitations. Individual patient data and individual indications for TOE by participating centers were not assessed which represents a limitation when interpreting associations, as a recall and an anchoring bias might be possible. Our data provide broad information on clinical routines and structural resources for performing TOE on German stroke units. We included stroke units of all levels of care and were able to achieve a response rate of > 70% and the participating centers, in their distribution, reflect the distribution of the German SUs according to certification levels. Therefore, our findings reflect general trends across Germany. Due to the nature of the study, we are unable to compare the structure of responding and non- responding stroke centers. Moreover, the responding leads provided estimates of TOE waiting times and given the nature of the study, we did not include individual patient data. Due to differences in other healthcare settings, the translation of our findings to other countries may be limited.

## Conclusion

Despite the limitations of self-reported estimates, the exploratory approach, and the lack of adjustment for confounders, our data provide valuable insights into clinical routines, structural resources and indications for performing TOE in hospitalized German stroke patients. Our analysis may facilitate the optimized use of TOE in hospitals and represent a first step towards the development of future guidelines and SOPs, in particular in the context of limited resources. By this, the care of this relevant group of patients could potentially be improved markedly.

## Electronic Supplementary Material

Supplementary Material 1

Supplementary Material 2

Supplementary Material 3

Supplementary Material 4

Supplementary Material 5

Supplementary Material 6

## Data Availability

The datasets used and/or analysed during the current study are available from the corresponding author on reasonable request.

## Abbreviations

AF: Atrial Fibrillation
AIS: Acute Ischemic Stroke
CRF: Case report files
DSG: German Stroke Society (Deutsche Schlaganfall-Gesellschaft)
PFO: Patent Foramen Ovale
SOP: Standard Operating Procedure
SPSS: Statistical Package for the Social Sciences
SU: Stroke Unit
TIA: Transient Ischemic Attack
TOE: Transoesophageal Echocardiography
TTE: Transthoracic Echocardiography
IQR: Interquartile Range
