# Supplementary material for: Clinical routines and structural resources for performing transoesophageal echocardiography on German stroke units
Source: Neurol Res Pract. 2026 May 19;8(1):41. doi: 10.1186/s42466-026-00500-9 (PMC13188604; doi:10.1186/s42466-026-00500-9)
Supplement: Supplementary file 1 — Supplementary Material 1 [file 42466_2026_500_MOESM1_ESM.docx]

**Supplementary Figure 1:** Stroke unit level of certification and waiting time for TOE: A) Grouped waiting times after request and certification levels, B) Number of treated patients (grouped) and waiting time after request

A) B)

Legend: TOE – transoesophageal echocardiography, SU – Stroke unit
