# Supplementary material for: Clinical routines and structural resources for performing transoesophageal echocardiography on German stroke units
Source: Neurol Res Pract. 2026 May 19;8(1):41. doi: 10.1186/s42466-026-00500-9 (PMC13188604; doi:10.1186/s42466-026-00500-9)
Supplement: Supplementary file 2 — Supplementary Material 2 [file 42466_2026_500_MOESM2_ESM.docx]

**Supplementary Figure 2:** Number of stroke unit beds and reports by leads on negotiations with TOE providers: With an escalating number of beds, it was increasingly often reported that the indication for TOE examinations often has to be negotiated vigorously with TOE providers (p=0.047, Table 2).

Legend: TOE – transoesophageal echocardiography
