# Supplementary material for: Clinical routines and structural resources for performing transoesophageal echocardiography on German stroke units
Source: Neurol Res Pract. 2026 May 19;8(1):41. doi: 10.1186/s42466-026-00500-9 (PMC13188604; doi:10.1186/s42466-026-00500-9)
Supplement: Supplementary file 3 — Supplementary Material 3 [file 42466_2026_500_MOESM3_ESM.docx]

**Supplementary Figure 3:** The spider chart shows the different constellations that impact individual decisions to perform TOE. For better clarity, the different constellations are divided into three separate graphs according to the questions B1 (top row, left), B2 (top row, right) and B3 (bottom) from the questionnaire


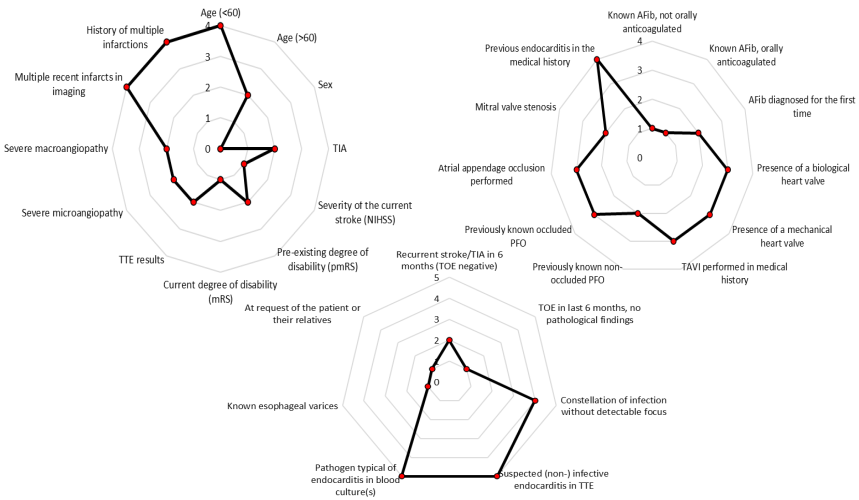


Legend: The value axis: the gradation of possible answers (0 – Never, 1 – Seldom, 2 – Sometimes, 3 – Frequently, 4 – Regularly, 5 – Always) and the given values represent the median for each respective category
