# Supplementary material for: Clinical routines and structural resources for performing transoesophageal echocardiography on German stroke units
Source: Neurol Res Pract. 2026 May 19;8(1):41. doi: 10.1186/s42466-026-00500-9 (PMC13188604; doi:10.1186/s42466-026-00500-9)
Supplement: Supplementary file 4 — Supplementary Material 4 [file 42466_2026_500_MOESM4_ESM.docx]

**Supplementary Table 4a:** Binary logistic regression between centers with a TOE rate <20% and centers with a TOE rate between 20% and 29%

| Variable | OR | 95%CI | p-value |
| --- | --- | --- | --- |
| Level of certification | 0.675 | 0.288 – 1.581 | 0.366 |
| Total number of ischemic strokes/TIA patients treated per year (2023) | 0.850 | 0.522 – 1.383 | 0.512 |
| Number of stroke unit beds | 0.905 | 0.518 – 1.581 | 0.727 |
| Unversity hospital | 1.667 | 0.457 – 6.076 | 0.439 |
| Presence of cardiology department | 1.437 | 0.273 – 7.574 | 0.669 |
| Types of units performing the TOE | 1.003 | 0.754 – 1.335 | 0.983 |
| Waiting time to TOE performance | 0.8 | 0.419 – 1.528 | 0.499 |

**Supplementary Table 4b:** Binary logistic regression between centers with a TOE rate <20% and centers with a TOE rate ≥30%

| Variable | OR | 95%CI | p-value |
| --- | --- | --- | --- |
| Level of certification | 0.610 | 0.256 – 1.452 | 0.264 |
| Total number of ischemic strokes/TIA patients treated per year (2023) | 0.716 | 0.435 – 1.180 | 0.190 |
| Number of stroke unit beds | 0.685 | 0.393 – 1.193 | 0.181 |
| Unversity hospital | 2.865 | 0.779 – 10.540 | 0.113 |
| Presence of cardiology department | 2.150 | 0.406 – 11.388 | 0.368 |
| Types of units performing the TOE | 0.852 | 0.626 – 1.160 | 0.310 |
| Waiting time to TOE performance | 1.026 | 0.562 – 1.872 | 0.934 |

**Supplementary Table 4c:** Binary logistic regression between centers with a TOE rate between 20% and 29% and centers with a TOE rate ≥30%

| Variable | OR | 95%CI | p-value |
| --- | --- | --- | --- |
| Level of certification | 0.945 | 0.413 – 2.160 | 0.893 |
| Total number of ischemic strokes/TIA patients treated per year (2023) | 0.944 | 0.559 – 1.592 | 0.828 |
| Number of stroke unit beds | 0.729 | 0.403 – 1.317 | 0.294 |
| Unversity hospital | 1.526 | 0.471 – 4.947 | 0.481 |
| Presence of cardiology department | 0.731 | 0.102 – 5.234 | 0.755 |
| Types of units performing the TOE | 0.821 | 0.585 – 1.153 | 0.256 |
| Waiting time to TOE performance | 1.093 | 0.512 – 1.954 | 0.763 |
