# Supplementary material for: Clinical routines and structural resources for performing transoesophageal echocardiography on German stroke units
Source: Neurol Res Pract. 2026 May 19;8(1):41. doi: 10.1186/s42466-026-00500-9 (PMC13188604; doi:10.1186/s42466-026-00500-9)
Supplement: Supplementary file 5 — Supplementary Material 5 [file 42466_2026_500_MOESM5_ESM.docx]

**Supplementary Table 5a:** Binary logistic regression between centers with a TOE waiting time from registration to performance of <24h and centers with a TOE waiting time of 24h to 48h

| Variable | OR | 95%CI | p-value |
| --- | --- | --- | --- |
| Level of certification | 0.492 | 0.232 – 1.045 | 0.065 |
| Total number of ischemic strokes/TIA patients treated per year (2023) | 0.991 | 0.605 – 1.623 | 0.970 |
| Number of stroke unit beds | 1.122 | 0.635 – 1.980 | 0.692 |
| Unversity hospital | 1.048 | 0.280 – 3.922 | 0.944 |
| Presence of cardiology department | 0.247 | 0.046 – 1.324 | 0.103 |
| Types of units performing the TOE | 0.807 | 0.606 – 1.075 | 0.142 |
| TOE rate | 0.782 | 0.504 – 1.214 | 0.273 |

**Supplementary Table 5b:** Binary logistic regression between centers with a TOE waiting time from registration to performance of 24h to 48h and centers with a TOE waiting time TOE waiting time >48h

| Variable | OR | 95%CI | p-value |
| --- | --- | --- | --- |
| Level of certification | 0.639 | 0.176 – 2.311 | 0.494 |
| Total number of ischemic strokes/TIA patients treated per year (2023) | 2.271 | 1.158 – 4.453 | **0.017** |
| Number of stroke unit beds | 0.663 | 0.331 – 1.330 | 0.247 |
| Unversity hospital | 1.403 | 0.412 – 4.779 | 0.589 |
| Presence of cardiology department | 0.854 | 0.063 – 11.658 | 0.906 |
| Types of units performing the TOE | 0.663 | 0.372 – 1.182 | 0.163 |
| TOE rate | 1.356 | 0.741 – 2.480 | 0.323 |

**Supplementary Table 5c:** Binary logistic regression between centers with a TOE waiting time from registration to performance of <24h and centers with a TOE waiting time >48h

| Variable | OR | 95%CI | p-value |
| --- | --- | --- | --- |
| Level of certification | 0.303 | 0.085 – 1.076 | 0.065 |
| Total number of ischemic strokes/TIA patients treated per year (2023) | 2.277 | 1.145 – 4.530 | **0.019** |
| Number of stroke unit beds | 0.877 | 0.433 – 1.779 | 0.716 |
| Unversity hospital | 2.128 | 0.316 – 14.323 | 0.438 |
| Presence of cardiology department | 0.186 | 0.01 – 3.432 | 0.258 |
| Types of units performing the TOE | 0.436 | 0.231 – 0.823 | **0.01** |
| TOE rate | 1.383 | 0.664 – 2.882 | 0.386 |
