# Supplementary material for: Clinical routines and structural resources for performing transoesophageal echocardiography on German stroke units
Source: Neurol Res Pract. 2026 May 19;8(1):41. doi: 10.1186/s42466-026-00500-9 (PMC13188604; doi:10.1186/s42466-026-00500-9)
Supplement: Supplementary file 6 — Supplementary Material 6 [file 42466_2026_500_MOESM6_ESM.docx]

| **Part A - General information about the stroke unit of your hospital** | | | | |
| --- | --- | --- | --- | --- |
| **A.1**  **What is the type of the stroke unit? (Level of certification)** (Please provide only one answer) | | | | |
| Supraregional stroke unit | | [ ] | Regional stroke unit | [ ] |
| Telemedically-connected stroke unit | | [ ] |  |  |
| **A.2. Total number of ischemic strokes/TIA treated at your stroke unit in 2023** (Please provide only one answer) | | | | |
| < 250 |  | [ ] | 750 - 999 | [ ] |
| 251 - 500 |  | [ ] | 1000 - 1250 | [ ] |
| 501 - 749 |  | [ ] | ≥ 1250 | [ ] |
| **A.3 Number of beds in your stroke unit** (Please provide only one answer) | | | | |
| ≤ 4 |  | [ ] | 9 - 12 | [ ] |
| 5 - 6 |  | [ ] | > 12 | [ ] |
| 7 - 8 |  | [ ] |  | |
| **A.4 Is it a university hospital?** (Please provide only one answer) | | | | |
| Yes |  | [ ] | No | [ ] |
| **A.5 Does your hospital have its own cardiology department?**  (Please provide only one answer) | | | | |
| Yes |  | [ ] | No | [ ] |
| **A.6 Transoesophageal echocardiography (TOE) examinations are performed at your clinic by:** (Please provide only one answer) | | | | |
| Cardiology department | | [ ] | Cardiologist on the stroke unit | [ ] |
| Medical practice or medical care center (MVZ) during the hospital stay | | [ ] | Medical practice or medical care center (MVZ) after discharge | [ ] |
| Attending physician | | [ ] | External medical practice or medical care center | [ ] |
| Medical practice or medical care center (MVZ) owned by the hospital | | [ ] |  | |

| **A.7 How long is the waiting time (in hours) from registration to performance of the TOE in your clinic? (estimated)** (Please provide only one answer) | | |  |
| --- | --- | --- | --- |
| < 12 h | [ ] | 24 – 48 h | [ ] |
| 12 – 24 h | [ ] | > 48 h | [ ] |
| **A.8 Does an in-house SOP exist for the indication of TOE examinations in stroke/TIA patients?** (Please provide only one answer) | | |  |
| Yes | [ ] | No | [ ] |
| **Free text for any comments (optional)** | | |  |
|  | | |  |

| **Part B - Information on the indication of TOE** | | | | | | |
| --- | --- | --- | --- | --- | --- | --- |
| **B.1 Which of the factors listed below influence your indication for a TOE?** (Please estimate; check only one option for each factor) | | | | | | |
|  | **Never (0%)** | **Sporadically**  **(<25%)** | **Occasionally**  **(25-50 %)** | **Frequently**  **(50-75%)** | **Regularly (75%-99%)** | **Always (100%)** |
| Age (<60) | [ ] | [ ] | [ ] | [ ] | [ ] | [ ] |
| Age (>60) | [ ] | [ ] | [ ] | [ ] | [ ] | [ ] |
| Sex | [ ] | [ ] | [ ] | [ ] | [ ] | [ ] |
| TIA | [ ] | [ ] | [ ] | [ ] | [ ] | [ ] |
| Severity of the current stroke (NIHSS) | [ ] | [ ] | [ ] | [ ] | [ ] | [ ] |
| Pre-existing degree of disability (pmRS) | [ ] | [ ] | [ ] | [ ] | [ ] | [ ] |
| Current degree of disability (mRS) | [ ] | [ ] | [ ] | [ ] | [ ] | [ ] |
| TTE results | [ ] | [ ] | [ ] | [ ] | [ ] | [ ] |
| Severe microangiopathy | [ ] | [ ] | [ ] | [ ] | [ ] | [ ] |
| Severe macroangiopathy | [ ] | [ ] | [ ] | [ ] | [ ] | [ ] |
| Multiple recent infarcts on imaging | [ ] | [ ] | [ ] | [ ] | [ ] | [ ] |
| History of multiple infarctions | [ ] | [ ] | [ ] | [ ] | [ ] | [ ] |

| **B.2 In what proportion of your stroke/TIA patients with known cardiac pathologies do you perform a TOE?** (Please estimate; check only one option for each factor) | | | | | | | | |
| --- | --- | --- | --- | --- | --- | --- | --- | --- |
|  | **Never (0%)** | **Sporadically**  **(<25%)** | **Occasionally**  **(25-50 %)** | **Frequently**  **(50-75%)** | | | **Regularly**  **(75-99%)** | **Always**  **(100%)** |
| Known atrial fibrillation, at onset of the current stroke not anticoagulated | [ ] | [ ] | [ ] | [ ] | | | [ ] | [ ] |
| Known atrial fibrillation, at onset of the current stroke anticoagulated | [ ] | [ ] | [ ] | [ ] | | | [ ] | [ ] |
| First diagnosis of atrial fibrillation during the current hospital stay | [ ] | [ ] | [ ] | [ ] | | | [ ] | [ ] |
| Presence of a biological heart valve replacement | [ ] | [ ] | [ ] | [ ] | | | [ ] | [ ] |
| Presence of a mechanical heart valve replacement | [ ] | [ ] | [ ] | [ ] | | | [ ] | [ ] |
| TAVI performed in medical history | [ ] | [ ] | [ ] | [ ] | | | [ ] | [ ] |
| Previously known non-occluded PFO | [ ] | [ ] | [ ] | [ ] | | | [ ] | [ ] |
| Previously known occluded PFO | [ ] | [ ] | [ ] | [ ] | | | [ ] | [ ] |
| Atrial appendage occlusion performed in medical history | [ ] | [ ] | [ ] | [ ] | | | [ ] | [ ] |
| Mitral valve stenosis | [ ] | [ ] | [ ] | [ ] | | | [ ] | [ ] |
| Previous endocarditis in medical history | [ ] | [ ] | [ ] | [ ] | | | [ ] | [ ] |
| **B.3 In what proportion of your stroke or TIA patients with the constellations listed below do you perform a TOE?** (Please estimate; check only one option for each factor) | | | | | | | | |
|  | **Never (0%)** | **Sporadically**  **(<25%)** | **Occasionally**  **(25-50 %)** | **Frequently**  **(50-75%)** | | | **Regularly**  **(75-99%)** | **Always (100%)** |
| Recurrent stroke/TIA within the last 6 months (TOE performed at previous event without pathological findings) | [ ] | [ ] | [ ] | [ ] | | | [ ] | [ ] |
| TOE performed in the last 6 months without pathological findings | [ ] | [ ] | [ ] | [ ] | | | [ ] | [ ] |
| Constellation of infection with clinical signs of infection, but no detectable focus | [ ] | [ ] | [ ] | [ ] | | | [ ] | [ ] |
| Suspicion of (non-) infective endocarditis in TTE | [ ] | [ ] | [ ] | [ ] | | | [ ] | [ ] |
| Detection of a pathogen, typical of infectious endocarditis in blood culture(s) | [ ] | [ ] | [ ] | [ ] | | | [ ] | [ ] |
| Known esophageal varices | [ ] | [ ] | [ ] | [ ] | | | [ ] | [ ] |
| At request of the patient or their relatives | [ ] | [ ] | [ ] | [ ] | | | [ ] | [ ] |
| **B.4 Are the TOE results obtained helpful for clinical decisions?** (Please provide only one answer) | | | | | | | | |
|  | **Never**  **(0%)** | **Sporadically (<25%)** | **Occasionally**  **(25-50 %)** | **Frequently**  **(50-75%)** | | | **Regularly**  **(75-99%)** | **Always (100%)** |
|  | [ ] | [ ] | [ ] | [ ] | | | [ ] | [ ] |
| **B.5** **The indication for TOE examinations often has to be negotiated vigorously with TOE providers in individual patients** (Please provide only one answer) | | | | | | | | |
| Yes | [ ] | | No | | | [ ] | | |
| **B.6 Do quantitative specifications (e.g., SU certification) influence your indication for a TOE?** (Please provide only one answer) | | | | | | | | |
| Yes | [ ] | | No | | | [ ] | | |
| **B.7 Please enter your TOE quota from 2023 (according to DSG, %)** | | | | | | | | |
| Ischemic  strokes | % | | TIAs | | % | | | |
| Spontaneous intracerebral hemorrhages | % | |  |  |  |  |  |  |
| **Free text for any comments (optional)** | | | | | | | | |
|  | | | | | | | | |

**Thank you very much for your time!**
